# SimpleTrackV2: Rethinking the Timing Characteristics for Multi-Object Tracking

**DOI:** 10.3390/s24186015

**Published:** 2024-09-17

**Authors:** Yan Ding, Yuchen Ling, Bozhi Zhang, Jiaxin Li, Lingxi Guo, Zhe Yang

**Affiliations:** 1 Key Laboratory of Dynamics and Control of Flight Vehicle, Ministry of Education, School of Aerospace Engineering, Beijing Institute of Technology, Beijing 100081, China; dingyan@bit.edu.cn (Y.D.); 3220220033@bit.edu.cn (Y.L.); 2 Science and Technology on Space Physics Laboratory, Beijing 100076, China; vpmaster2@gmail.com (J.L.); guolingxi@126.com (L.G.); yangzh_calt@163.com (Z.Y.)

**Keywords:** multiple object tracking, timing characteristics, state prediction, state fusion

## Abstract

Multi-object tracking tasks aim to assign unique trajectory codes to targets in video frames. Most detection-based tracking methods use Kalman filtering algorithms for trajectory prediction, directly utilizing associated target features for trajectory updates. However, this approach often fails, with camera jitter and transient target loss in real-world scenarios. This paper rethinks state prediction and fusion based on target temporal features to address these issues and proposes the SimpleTrackV2 algorithm, building on the previously designed SimpleTrack. Firstly, to address the poor prediction performance of linear motion models in complex scenes, we designed a target state prediction algorithm called LSTM-MP, based on long short-term memory (LSTM). This algorithm encodes the target’s historical motion information using LSTM and decodes it with a multilayer perceptron (MLP) to achieve target state prediction. Secondly, to mitigate the effect of occlusion on target state saliency, we designed a spatiotemporal attention-based target appearance feature fusion (TSA-FF) target state fusion algorithm based on the attention mechanism. TSA-FF calculates adaptive fusion coefficients to enhance target state fusion, thereby improving the accuracy of subsequent data association. To demonstrate the effectiveness of the proposed method, we compared SimpleTrackV2 with the baseline model SimpleTrack on the MOT17 dataset. We also conducted ablation experiments on TSA-FF and LSTM-MP for SimpleTrackV2, exploring the optimal number of fusion frames and the impact of different loss functions on model performance. The experimental results show that SimpleTrackV2 handles camera jitter and target occlusion better, achieving improvements of 1.6%, 3.2%, and 6.1% in MOTA, IDF1, and HOTA, respectively, compared to the SimpleTrack algorithm.

## 1. Introduction

Multi-object tracking is a challenging task in computer vision that aims to estimate the position and identity of multiple objects in a video sequence [1,2]. Detection-based tracking is a common paradigm for these tasks. It completely decouples the object detection process from the multi-object tracking process. As a result, the tracking process can be viewed as a post-processing step of the detection results. This separation allows for independent optimization of detection and tracking algorithms.

In target-intensive scenarios, tracking becomes challenging due to occlusion between targets and irregular camera jitter caused by external forces (such as wind or mechanical vibrations). When tracking relies solely on the position information the detector provides, it often leads to frequent jumps in the target’s identity. To address this issue, a target reidentification feature is introduced after detection to mitigate environmental interference. In this paper, the “state” in target state prediction refers to the target’s position, represented as (x, y, w, h) in a 2D image. This includes the pixel coordinates of the target’s center relative to the upper-left corner of the image, along with the width and height of the detection frame. On the other hand, the “state” in target state fusion refers to the target’s reidentification feature, which is typically a high-dimensional vector containing more identity information than the position feature.

In recent years, many detection-tracking paradigms [3,4,5,6,7,8] have relied on a combination of target state prediction from the previous frame and target state detection from the current frame to determine the state of the target. The results of the target state fusion of the prior frame are then correlated with the target state of the current frame, allowing for updates to the trajectory encoded with the corresponding identity. The Kalman filter algorithm is the most commonly used method for target state prediction. For target state fusion, the exponential sliding average fusion algorithm is frequently employed, or alternatively, the state of the current frame is used directly as the latest state of the trajectory [9,10,11,12,13]. We found several limitations upon re-evaluating the state prediction and fusion methods in detection-tracking paradigms. Existing target state prediction methods often perform poorly in complex scenes, such as those with frequent occlusions between targets or camera jitter. The Kalman filter motion prediction in particular shows significant bias when the target’s prior knowledge is missing or inaccurate. Moreover, most existing target state fusion methods focus on spatial relationships between different state cues, with limited consideration of temporal dimension fusion. According to our experiments, the performance of these methods degrades significantly in environments with frequent target occlusions.

**Figure 1:**
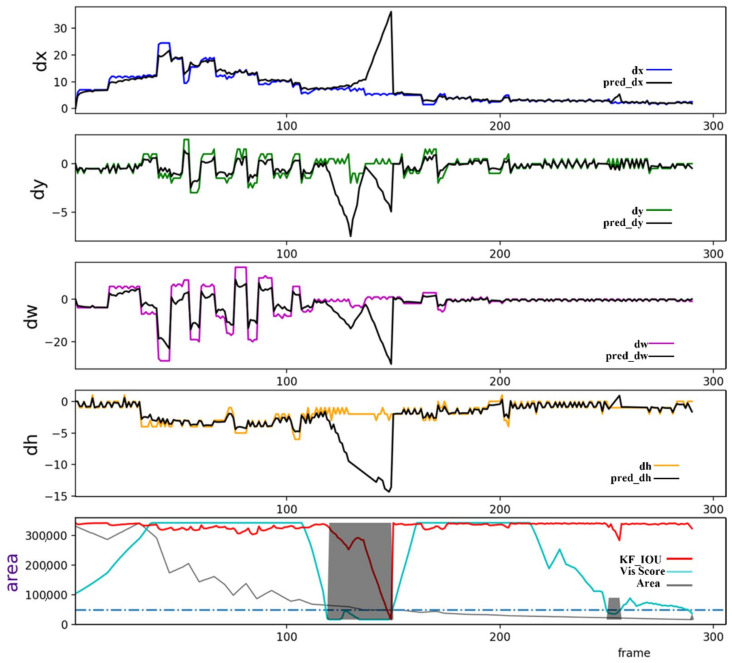
Motion state differences and linear Kalman filter predictions for the pedestrian target with video sequence ID 23 in MOT17-09. The linear Kalman filter predicts the center point coordinates and the width and height of the target’s bounding box based on a linear motion model. The black curve represents the frame-to-frame differences in the Kalman filter’s predictions, the blue and green curves show the differences in the horizontal and vertical coordinates of the target’s center point, respectively, the purple and orange curves illustrate the differences in the target’s width and height, and the red and cyan curves depict the intersection over union (IoU) of the predicted bounding boxes and the degree of target occlusion.

In pedestrian-dense scenes, we analyzed the difference between the target’s motion state in consecutive frames and the prediction results obtained using a linear Kalman filter. The analysis results are presented in Figure 1.

Before the 100th frame, when the target is not yet occluded, the Kalman filter’s predictions closely match the actual results, and the calculated intersection over union (IoU) between consecutive frames remains consistently high. This high IoU supports accurate identity assignment during subsequent target association. However, starting from frame 100, as the target begins to experience occlusion, the absence of new detection boxes for updating the Kalman filter causes its predictions to deviate from the true trajectory gradually. This deviation eventually leads to errors in the target’s identity assignment. Thus, it can be concluded that the linear Kalman filter has limited accuracy when predicting target motion states over extended periods. Furthermore, Figure 1 shows that during pedestrian movement, there is a periodic swinging of the arms and changes in stride. This causes the frame difference data for the pedestrian’s motion state to exhibit distinct periodic characteristics.

To address this problem, we developed an LSTM-MP model to extract temporal features of the target state for improved prediction. We further incorporated an attention mechanism and designed a spatiotemporal attention-based target appearance feature fusion (TSA-FF) algorithm to compute fusion coefficients from these temporal features. To demonstrate the effectiveness of our proposed prediction and fusion methods, we integrated them into the SimpleTrack tracking framework [1]. As shown in Section 4.3, our approach improved multiple MOT metrics, including MOTA, IDF1, and HOTA.

To further explore the advantages of target state prediction and fusion methods based on temporal features, we propose the SimpleTrackV2 tracking framework, an extension of SimpleTrack. This framework uses a two-stage network to acquire the target state: YOLOX [14] extracts positional features, while SBSResNest [15] captures appearance features. SimpleTrackV2 incorporates a new target state fusion mechanism, TSA-FF, and a novel LSTM-based target state prediction model, LSTM-MP. Experimental results demonstrate that SimpleTrackV2 performs better than the original SimpleTrack algorithm in terms of MOTA, IDF1, and HOTA.

Our main contributions are as follows.

1. We propose a new target state prediction model, LSTM-MP, which leverages the historical information of the target state to enhance prediction accuracy. This model remains effective even when state observations are correct or prior knowledge of the target is missing or inaccurate, improving the algorithm’s tracking robustness.
2. We design a novel target state fusion algorithm, TSA-FF, which incorporates both temporal and spatial attention mechanisms. This approach enhances the ability to distinguish target identities, providing more reliable state information for the subsequent data association stage.

The remainder of the paper is arranged as follows. Section 2 summarizes related work, including target reidentification feature extraction, target state prediction, and target state fusion. Section 3 introduces the methodology of SimpleTrackV2, including the target state prediction model LSTM-MP and the target state fusion model TSA-FF. In Section 4, experimental results are provided to verify the performance of the proposed SimpleTrackV2. Section 5 briefly summarizes the work and discusses future directions.

## 2. Related Work

Multi-target tracking technology primarily involves target detection, feature extraction, motion prediction, data association, and trajectory management. The target state prediction method introduced in this paper can be considered an approach for motion prediction, while target state fusion serves as an enhancement to target reidentification features. Therefore, this paper reviews relevant research in three key areas: target reidentification feature extraction, target state prediction, and target state fusion.

### 2.1. Target Reidentification Feature Extraction

Target reidentification feature extraction seeks to obtain robust target features under varying observation viewpoints, image resolutions, lighting conditions, poses, occlusions, modalities, camera environments, and backgrounds [16,17,18,19,20,21,22]. These models typically treat the same objects in different frames as distinct classes and learn embeddings of cropped detection images using cross-entropy loss or triplet loss for ID classification [19,23,24,25,26,27,28,29,30]. Many algorithms incorporate additional modules in detection tracking paradigms to post-process network inference results. For example, the MOT with multiple cues algorithm employs GoogLeNet to extract reidentification features and then uses the SAC module for classification [31]. Similarly, the multiplex labeling graph MOT algorithm uses a detection multiplexing method to handle target reidentification features and address occlusion issues [32]. While these methods improve performance on specific datasets, they also introduce significant computational overhead, increasing execution time and limiting the model’s portability and reusability.

To address this problem, SimpleTrackV2 employs the FastReID [15] framework to construct a target reidentification feature extraction network and directly uses its outputs for subsequent state fusion. FastReID provides a modular framework for end-to-end feature extraction in ReID tasks. Unlike traditional applications of FastReID, which focus solely on reidentification, SimpleTrackV2 leverages these features to improve multi-target tracking. By using target reidentification features as the foundation for state fusion, SimpleTrackV2 enhances overall tracking performance.

### 2.2. Target State Prediction Methods

Most multi-target tracking algorithms use a linear motion model to predict the target state [9,10,11,12,25,33,34,35,36,37,38]. However, this approach assumes linear target motion and struggles to predict the target’s subsequent state during nonlinear motion accurately.

SimpleTrackV2 incorporates the LSTM-MP model to address this limitation, leveraging the long short-term memory (LSTM) network’s ability to capture and maintain information over long time spans [39,40,41,42,43,44,45]. Unlike the linear motion model, LSTM-MP integrates the temporal characteristics of the target state to better model its motion patterns, providing significantly greater robustness.

### 2.3. Target State Fusion Methods

Data association based on target states is typically performed using single-frame target states [11,12,46,47,48,49,50]. Methods like DeepSORT [12] and ByteTrack [13] associate the target state of the previous frame with the current frame. While these approaches have relatively simple pipelines, they struggle to produce accurate results during occlusion when relying solely on single-frame target states.

BoT-SORT [9] sought to address this issue, employing an exponential averaging method to determine state fusion coefficients. However, in complex scenarios, the importance of target states does not always follow a simple exponential relationship with time. Unlike BoT-SORT, SimpleTrackV2 uses the TSA-FF algorithm to compute state fusion coefficients based on the temporal characteristics of target states. This approach allows SimpleTrackV2 to model the fusion mechanism of target states over time, achieving stable data association.

## 3. SimpleTrackV2

**Figure 2:**
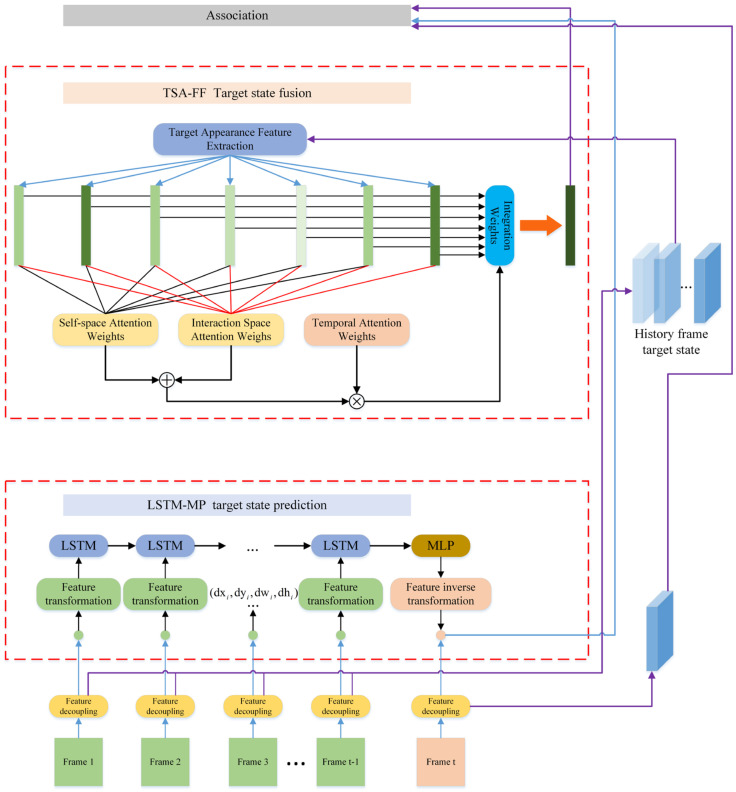
The target state prediction and fusion pipeline in SimpleTrackV2. SimpleTrackV2 inherits the feature decoupling and association components from SimpleTrack, with the improvements highlighted in the red boxes, which primarily include the design of LSTM-MP for target state prediction and TSA-FF for target state fusion. In the diagram, the blue output lines from feature decoupling represent the extracted positional features, while the purple arrows indicate the extracted appearance features. The LSTM-MP model utilizes the differences in the target’s motion state between consecutive frames as input to predict future motion states. The TSA-FF algorithm computes temporal and spatial attention weights to determine fusion weight coefficients, facilitating the effective fusion of target states.

In this section, we present the details of SimpleTrackV2. As illustrated below, SimpleTrackV2 inherits the feature decoupling and association components from SimpleTrack. The improvements introduced in SimpleTrackV2, highlighted by the red boxes in Figure 2, include the design of LSTM-MP for target state prediction and the spatiotemporal attention-based target appearance feature fusion (TSA-FF) algorithm for target state fusion.

### 3.1. Target State Prediction

Current multi-target tracking algorithms typically employ a linear motion model, with the target’s motion state modeled using a linear Kalman filter. During tracking, a Kalman filter is initialized for each target. Before the target association begins, the Kalman filter predicts the target’s position in the next frame. After completing the target association, the filter is updated using information from the matched detection frame. The target state ${\hat{x}}_{k}$ of the current frame can be obtained from the target state ${{\hat{x}}^{\prime }}_{k-1}$ of the previous frame, and the process can be expressed as:

(1) $${\hat{x}}_{k}=F_{k}{{\hat{x}}^{\prime }}_{k-1}+B_{k}{\vec{u}}_{k}$$

where F denotes the state transfer matrix, B denotes the control matrix, and ${\vec{u}}_{k}$ denotes the control input.

The process of predicting the error covariance $P_{k}$ of the current time frame based on the error covariance of the target state ${P^{\prime }}_{k-1}$ in the previous frame and the process noise $Q_{k}$ can be expressed as:

(2) $$P_{k}=F_{k}{P^{\prime }}_{k-1}F_{k}^{T}+Q_{k}$$

In the update phase, the predicted error covariance $P_{k}$ and measurement noise $R_{k}$ are first used to calculate the Kalman gain $K^{\prime }$, which can be expressed as:

(3) $$K^{\prime }=P_{k}H_{k}^{T}{\left(H_{k}P_{k}H_{k}^{T}+R_{k}\right)}^{-1}$$

where H is the observation matrix that describes the observation model.

Updating the state estimate for the current frame based on the observed and predicted states can be expressed as:

(4) $${\hat{x}}_{k}^{\prime }={\hat{x}}_{k}+K^{\prime }({\vec{z}}_{k}-H_{k}{\hat{x}}_{k})$$

where ${\hat{x}}_{k}^{\prime }$ denotes the updated target state of the current frame and ${\vec{z}}_{k}$ denotes the observed value of the target of the current frame.

Finally, the error covariance $P_{k}^{\prime }$ of the current frame is updated based on the Kalman gain $K^{\prime }$, which can be expressed as:

(5) $$P_{k}^{\prime }=P_{k}-K^{\prime }H_{k}P_{k}$$

The process noise Q in the Kalman filtering algorithm reflects the uncertainty in the system model, with a larger Q indicating greater uncertainty in the system model, and the measurement noise covariance matrix R reflects the uncertainty in the sensor measurements, with a large R indicating greater measurement uncertainty. The dependence of the filter on the measured and predicted values can be adjusted by varying the size of Q and R. Although it is possible to mitigate the effect of the predicted values on the filter by reducing Q, this approach also suffers from the problem of not being able to correct by the predicted values when there is a large uncertainty in the detected values, and also the adjustment of Q and R needs to be continually experimented with.

**Figure 3:**
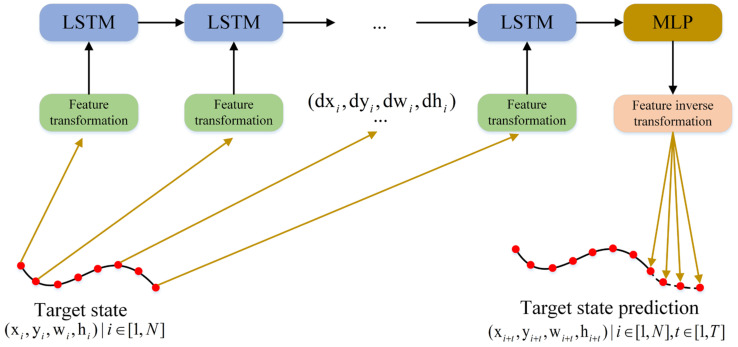
The LSTM-MP model begins by applying a feature transformation method to convert the motion features of the target’s historical frames into the difference between its front and back frame motion states. This difference is then used as input to the model. The LSTM-MP model encodes and reduces the dimensionality of these features through a multilayer perceptron (MLP) network. Finally, an inverse feature transformation is applied to predict the target’s future motion state, thereby achieving effective motion state prediction.

To address the above problems, this paper designs an LSTM-MP module based on the LSTM structure. The LSTM-MP uses the LSTM as an encoder and the MLP as a decoder to predict the target’s motion, as shown in Figure 3.

**Figure 4:**
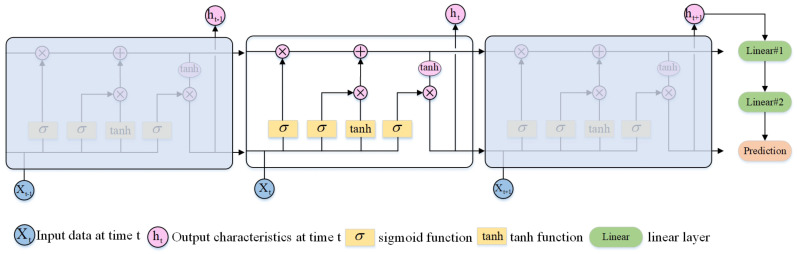
The overall structure of the LSTM-MP model.

The overall structure of LSTM-MP is shown in Figure 4. The encoder uses an LSTM network, and the decoder uses an MLP (multilayer perceptron) network, where the LSTM of the encoder consists of an oblivion gate, a memory gate, a cellular state, and an output gate.

The input of the forgetting gate consists of the target state $x_{t}$ of the current frame and the $h_{t-1}$ of the target state of the previous frame computed by the LSTM-MP, and the output of the forgetting gate can be expressed as:

(6) $$f_{t}=\sigma (W_{f}h_{t-1}+U_{f}x_{t-1}+b_{f})$$

where $f_{t}$ denotes the moment t oblivion gate output, σ(·) denotes the sigmoid function, $W_{f}$ and $U_{f}$ denote the weight parameters of the oblivion gate, and b denotes the bias parameter of the oblivion gate.

LSTM-MP has two memory gates, which use sigmoid and tanh functions to compute the memorization degree of different data, respectively. The data computation of the two memory gates can be expressed as:

(7) $$i_{t}=\sigma (W_{i}h_{t-1}+U_{i}x_{t}+b_{i})$$

(8) $$a_{t}=\tanh(W_{a}h_{t-1}+U_{a}x_{t}+b_{a})$$

where $i_{t}$ and $a_{t}$ denote the memory gate output at moment *t*, σ(·) denotes the sigmoid function, tanh(·) denotes the tanh function, $W_{i}$, $U_{i}$, $W_{a}$, and $U_{a}$ denote the weight parameter of the memory gate input data, and $b_{i}$ and $b_{a}$ denote the bias parameter of the memory gate input data, respectively.

The cell state $C_{t}$ in the LSTM-MP model denotes the memory state of the encoder for the data at moment t, which can be represented by a combination of the forgetting gate and memory gate:

(9) $$C_{t}=C_{t-1}⊙f_{t}+i_{t}⊙a_{t}$$

where ⊙ denotes the Hadamard product, i.e., element-by-element multiplication.

The output gate $h_{t}$ in the encoder is computed from the combination of the cell state $C_{t}$, the output state $h_{t-1}$ of the previous frame, and the input combination $x_{t}$ of the current frame, and the operation can be expressed as:

(10) $$h_{t}=o_{t}⊙\tanh(C_{t})$$

(11) $$o_{t}=\sigma (W_{o}h_{t-1}+U_{o}x_{t}+b_{o})$$

The LSTM-MP model linearly maps the output of the last frame of data of the LSTM structure and computes the final predicted output through two linear layers. The parameters of the designed linear layers are shown in Table 1, where the output dimension of Linear#2 is 20, which indicates the prediction of the next five frames of target motion data. The length of each data frame is 4, which indicates the prediction parameters of the target’s center point position, width, and height.

### 3.2. Target State Fusion

Traditional methods for fusing appearance features over time assume that more recent target information is more valuable, modeling fusion coefficients as an exponential function of the time interval between the historical and current frames. However, in complex scenarios, the relationship between a target’s appearance features across different time points is more complex than an exponential function can capture. Thus, the fusion coefficient should account not only for the time interval but also for the information extracted from the appearance features. Our approach enhances the appearance features of the target in the current frame, improving the differentiation between similar targets and maintaining feature consistency for the same target during tracking.

**Figure 5:**
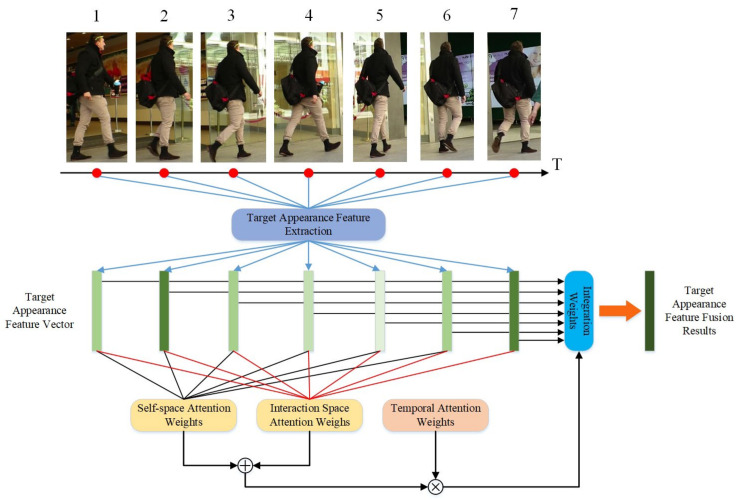
The TSA-FF algorithm first computes the temporal and spatial attention weights for the appearance feature vectors of different time frames for the same target. It then sums the self-spatial attention coefficients of each historical frame with the interaction spatial attention coefficients and multiplies this sum by the temporal attention weights to determine the final fusion coefficients. Finally, the appearance features of each historical frame are weighted and averaged using these fusion coefficients, followed by normalization to obtain the fused target appearance features.

To capture the state-implicit correlations of the target’s historical frames, we propose a spatiotemporal attention-based target appearance feature fusion (TSA-FF) algorithm, which computes adaptive fusion coefficients. TSA-FF combines historical state information to enhance the robustness of the current frame states. The algorithm employs an attention mechanism to calculate weighted fusion coefficients for target appearance features, as illustrated in Figure 5. Given the challenges posed by complex backgrounds and target motion, TSA-FF incorporates features specific to multi-target tracking and introduces self-spatial and interactive spatial attention weights within the attention mechanism.

The self-spatial attention weighting evaluates the fusion weights of appearance features from different historical frames by calculating their similarity. If a historical frame has a higher similarity to all other historical frames, it is considered a key frame and is assigned a greater fusion weight. The process can be represented as:

(12) $$attention_{self}^{n\times n}=f_{history}^{n\times d}⊗{f_{history}^{n\times d}}^{T}$$

(13) $$w_{s-self}^{n\times 1}=mean(attention_{history}^{n\times n})$$

where $attention_{self}^{n\times n}$ denotes the self-attention among the target history frame appearance features, $f_{history}^{n\times d}$ denotes the number of history frames, d denotes the target appearance feature dimensions, and ⊗ denotes matrix multiplication. $w_{s-self}^{n\times 1}$ denotes the self-spatial attention weight, and mean(·) denotes the mean of the matrix by row.

To extract the degree of influence that appearance features of a target’s history frames have on the current frame, we propose the concept of interaction space attention weight. This method involves calculating the similarity coefficient between the appearance features of the target’s current frame and those of all its history frames.

Interactive spatial attention weighting uses this similarity coefficient to determine the interactive spatial attention fusion weights of the history frames, a process that can be expressed as:

(14) $$attention_{cross}^{1\times n}=f_{current}^{1\times d}⊗{f_{history}^{n\times d}}^{T}$$

(15) $$w_{s-cross}^{n\times 1}={\left(attention_{cross}^{1\times n}\right)}^{T}$$

where $attention_{cross}^{1\times n}$ denotes the interaction attention between the appearance features of the target’s current frame and the history frames, $f_{current}^{1\times d}$ denotes the appearance features of the target’s current frame, $f_{history}^{n\times d}$ denotes the appearance features of the target’s history frames, n denotes the number of history frames, d denotes the dimension of the target’s appearance features, ⊗ denotes the matrix multiplication, and $w_{s-cross}^{n\times 1}$ denotes the interaction spatial attention weight. If a history frame has a higher similarity to the target’s current frame, it is considered to contribute more to feature fusion. Consequently, it is assigned a larger fusion weight.

Instead of modeling time intervals through manual design, self-space attention weights and interaction space attention weights extract complex temporal dependencies from the perspective of target historical appearance features in higher dimensions. This approach determines the temporal fusion method of target appearance features in complex scenes. Based on self-space attention weights and interaction space attention weights, the target appearance feature fusion weights are represented as:

(16) $$w_{combine}^{n\times 1}=(w_{s-cross}^{n\times 1}+w_{s-self}^{n\times 1})⊙w_{t}^{n\times 1}$$

where ⊙ denotes the Hadamard product, i.e., the multiplication of elements between matrices. $w_{combine}^{n\times 1}$ denotes the fusion weight result, obtained by summing the self-space attention weights and the interaction space attention weights, followed by an arithmetic operation with the temporal attention weights.

Further, the fused target appearance feature may be represented as:

(17) $$f_{combine}^{1\times d}=norm(norm({w_{combine}^{n\times 1}}^{T}⊗f_{history}^{n\times d})+f_{current}^{1\times d})$$

The fused target appearance feature $f_{combine}^{1\times d}$ contains the appearance feature information $f_{history}^{n\times d}$ of the target in the previous n frames. The attention weight $w_{combine}^{n\times 1}$ is no longer the traditional weight designed based on artificial a priori knowledge, but the result of the combined consideration of self-space attention weight and interaction space attention weight, and norm(·) denotes the feature vector normalization operation.

## 4. Experiments

### 4.1. Datasets and Metrics

#### 4.1.1. Datasets

We validate the proposed algorithm using the MOTChallenge2017 pedestrian multi-target tracking dataset, which consists of 14 video sequences, 7 for training and 7 for testing. For experimental purposes, each training sequence is divided in half, with the first half used for training and the second half for validation. This dataset poses significant challenges to tracking performance due to its high crowd density, diverse environmental conditions, and varying camera perspectives.

#### 4.1.2. Evaluation Metrics

To evaluate tracking performance, we employed TrackEval to assess all metrics, including MOTA [51], IDF1 [52], and HOTA [53]. MOTA is calculated based on false positives (FPs), false negatives (FNs), and identity switches (IDs), primarily reflecting detection performance, as the number of FPs and FNs exceeds that of IDs. IDF1 measures the capacity for identity retention, focusing more on association performance. HOTA, a metric introduced recently, explicitly balances the effectiveness of accurate detection, association, and localization [13].

### 4.2. Implementation Details

#### 4.2.1. Tracker

We used SimpleTrack [1] as the baseline model, and in the tracking phase, SimpleTrack defaults to a high detection score threshold $\tau_{\mathrm{high}}$ of 0.3, a low detection score threshold $\tau_{\mathrm{low}}$ of 0.2, a trajectory initialization score ε of 0.6, and a trajectory retrieval score $\varepsilon_{r}$ of 0.1. In the linear assignment step, the assignment threshold is 0.8 for high-confidence detection and 0.4 for low-confidence detection.

#### 4.2.2. Target Motion Trajectory Dataset Construction

The target motion state prediction model, LSTM-MP, requires the target’s historical frame motion states as inputs and uses the future frame motion states as supervised data for training. We construct the target motion trajectory dataset from the MOT17 validation set. Ground truth annotations are first loaded from the MOT17 dataset. For each video sequence, we extracted all motion states of targets with the same identity ID, computed the frame differences between consecutive motion states, arranged them chronologically, and saved the results for each identity ID.

We implemented a data loading strategy to train the model, which handles varying lengths of historical frame inputs and future frame outputs, as outlined in Algorithm 1.
**Algorithm:** Target state prediction model training loading data strategy
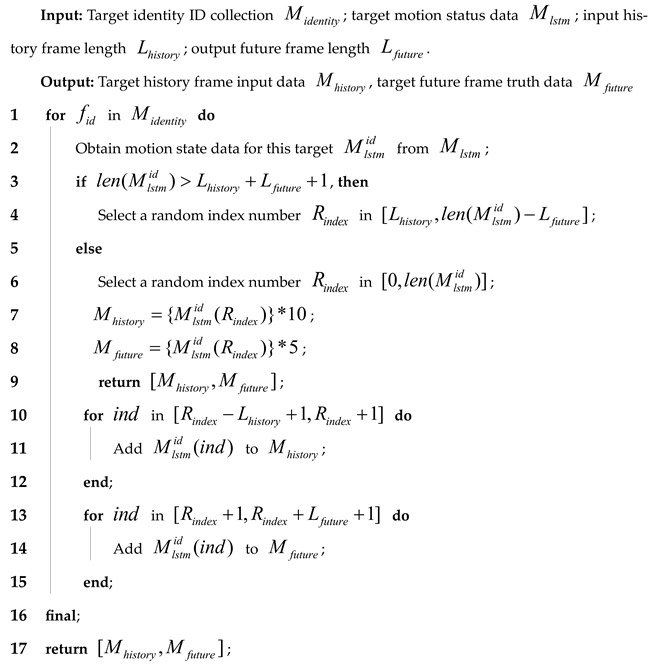


### 4.3. Ablation Studies

#### 4.3.1. Comparison with Other Methods

We used the team’s previous work, SimpleTrack [1], as a baseline model, which has demonstrated superior performance compared to algorithms such as RelationTrack [54], Semi-TCL [29], CSTrack [55], FairMOT [10], TransTrack [56], and others. As shown in Table 2, due to the overprediction of densely packed small targets in the MOT17 dataset and the mutual suppression caused by the joint query mechanism for tracking and detection in the transformer decoder, especially when new targets are close to already tracked ones, this conflict limits the ability of Transformer-based methods to detect new targets. Our method, SimpleTrackV2, employs the JDE architecture, integrating detection and feature extraction into a single network while decoupling them from subsequent motion prediction and data association. Leveraging the temporal memory mechanism of the LSTM-MP model and the effective feature enhancement capability of the TSA-FF, SimpleTrackV2 achieves higher detection and tracking performance. It surpasses SimpleTrack by 6.1% in HOTA, 3.2% in IDF1, and 1.6% in MOTA. These experimental results further validate the effectiveness of our proposed method.

#### 4.3.2. Target Appearance Feature Fusion Model Ablation Experiment

We compared the performance of single-frame appearance features without fusion, the exponential sliding average fusion method, and the spatiotemporal attention feature fusion method on the MOT17 dataset. The evaluation metrics used were MOTA, IDF1, and HOTA. The results are shown in Table 3.

Our results indicate that the exponential sliding average method has no significant advantage over single-frame appearance features, outperforming them by only 0.004 percentage points in the MOTA metric.

In contrast, our proposed appearance feature fusion method based on spatiotemporal attention demonstrates higher tracking accuracy and stability. Compared to the exponential sliding average method, our feature fusion method improves the MOTA metric by 0.34 percentage points, the IDF1 metric by 0.85 percentage points, and the HOTA metric by 0.289 percentage points.

#### 4.3.3. Analysis of Fusion Frame Count in the Target Appearance Feature Fusion Model

To verify the effect of different numbers of history frames on the fusion of target appearance features, we tested the tracking performance of the model under various conditions: fusing 10, 20, 30, 40, and 50 history frames. The results are summarized in Table 4.

The results indicate that the tracking performance improves gradually as the number of fused history frames increases, reaching its peak when fusing 40 history frames. At this point, the tracking algorithm achieves an MOTA metric of 76.872, an IDF1 metric of 79.097, and an HOTA metric of 67.528.

#### 4.3.4. Comparative Experiments on Target Motion State Prediction Models

To evaluate the effectiveness of LSTM-MP, we used SimpleTrack [1] as the baseline model. The appearance feature fusion algorithms utilize a spatiotemporal attention fusion method with 40 historical frames. We compared the tracking performance of the LSTM-MP algorithm using two different input configurations, as presented in Table 5.

Configuration #1: The inputs to the target motion state prediction network include the target center point coordinates, target width and height, and their respective frame differences, totaling 8 data points.

Configuration #2: The inputs consist of only the frame differences of the target center point coordinates and width–height, totaling 4 data points.

For both configurations, the outputs are the center point and width–height frame differences of the target’s future frames. The results show that LSTM-MP #2 outperforms LSTM-MP #1, achieving a 0.402 percentage point increase in the MOTA metric, a 0.762 percentage point increase in the IDF1 metric, and a 0.843 percentage point increase in the HOTA metric.

Due to the different supervisory effects of various loss functions on LSTM-MP training, we compared the training results of LSTM-MP models under the supervision of the Smooth L1 and MSE loss functions. The results are presented in the following table. The findings indicate that, under the supervision of the MSE loss function, the LSTM-MP #2 model achieves higher MOTA and IDF1 metrics compared to the Smooth L1 loss function. However, the HOTA metric is slightly lower for the MSE-trained model. The results are shown in Table 6.

To assess the effectiveness of LSTM-MP in enhancing tracking performance, we compared it with the GRU (gated recurrent unit) and Kalman filter algorithms. The experimental results are presented in Table 7. The GRU model performed worse in all tracking metrics compared to the LSTM-based model. The LSTM-MP + KF model, which employs LSTM for motion prediction in mobile camera scenarios and Kalman filtering for fixed camera scenarios, outperformed the Kalman filter alone in terms of MOTA, IDF1, and HOTA. However, due to the additional computational overhead introduced by LSTM-MP, the algorithm’s runtime efficiency decreases by 5.36 fps, resulting in a final performance of 12.44 fps.

## 5. Conclusions and Future Work

We propose an adaptive target state fusion method, spatiotemporal attention-based target appearance feature fusion (TSA-FF), within the SimpleTrack framework. TSA-FF utilizes historical state information to compute fusion coefficients, mitigating the impact of occlusion on target state estimation. Additionally, we introduce a target state prediction module, LSTM-MP, to model nonlinear motion patterns of the target. LSTM, a specialized recurrent neural network (RNN), excels at capturing and retaining dependencies within long sequences. Its unique memory cells effectively manage the flow of past information, allowing LSTM to leverage historical motion states when predicting future states, critical for multi-object tracking under occlusion and jitter. LSTM’s ability to learn nonlinear patterns in data enables more accurate predictions of future target positions, particularly in scenarios involving complex and nonlinear motion. This stands in contrast to linear Kalman filtering (LKF), which relies on process noise models and can misinterpret irregular or sudden jitters, leading to prediction errors. Finally, as a data-driven model, LSTM can autonomously adapt to various complex scenarios through extensive training, unlike LKF, which is limited by its reliance on preset models and noise parameters. Together, these components constitute SimpleTrackV2, which achieves 67.7 HOTA and 76.9 MOTA on the MOT17 dataset, surpassing SimpleTrack’s performance of 61.6 HOTA and 76.3 MOTA, and demonstrating superior adaptability and robustness in dynamic tracking environments.

Future work will focus on reducing the space complexity of the target state fusion algorithm and enhancing the generalization capabilities of the state prediction module.

## Figures and Tables

**Table 1:** Decoder MLP parameter setting.

Linear Layer	Input Dimension	Output Dimension
Linear#1	128	64
Linear#2	64	20

**Table 2:** Comparison with existing methods. The symbol “↑” indicates that the higher the value of the metric, the better the tracking performance of the algorithm.

Tracker	HOTA↑	IDF1↑	MOTA↑
SimpleTrack [1]	61.6	76.3	75.3
FairMOT [10]	59.3	72.3	73.7
Semi-TCL [29]	59.8	73.2	73.3
RelationTrack [54]	61.0	74.7	73.8
CSTrack [55]	59.3	72.6	74.9
TransTrack [56]	54.1	63.5	75.2
MeMOTR [57]	58.8	71.5	72.8
GTR [58]	59.1	71.5	75.3
SimpleTrackV2	67.7	79.5	76.9

**Table 3:** We conducted ablation experiments on the target state fusion model, comparing state fusion methods using only single-frame states, exponential sliding average fusion, and the TSA-FF fusion algorithm proposed in this paper. The symbol “↑” indicates that the higher the value of the metric, the better the tracking performance of the algorithm.

Tracker	State Fusion Method	MOTA↑	IDF1↑	HOTA↑
SimpleTrackV2	Single Frame	76.528	78.247	67.239
	EMA	76.532	78.247	67.239
	TSA-FF	76.872	79.097	67.528

**Table 4:** Hyperparameter experiments with TSA-FF in MOT17, where the number following TSA-FF indicates the number of historical frames fused. The symbol “↑” indicates that the higher the value of the metric, the better the tracking performance of the algorithm.

Tracker	Fusion Frames	MOTA↑	IDF1↑	HOTA↑
SimpleTrackV2	TSA-FF (10)	76.521	78.318	67.309
	TSA-FF (20)	76.51	78.323	67.308
	TSA-FF (30)	76.523	78.341	67.314
	TSA-FF (40)	76.872	79.097	67.528
	TSA-FF (50)	76.867	79.094	67.527

**Table 5:** Performance of LSTM-MP tracking with different input data. The symbol “↑” indicates that the higher the value of the metric, the better the tracking performance of the algorithm.

State Fusion	State Prediction	MOTA↑	IDF1↑	HOTA↑
TSA-FF (40)	LSTM-MP #1	75.684	77.414	65.664
	LSTM-MP #2	76.086	78.176	66.507

**Table 6:** Performance of LSTM-MP tracking with different loss function supervision. The symbol “↑” indicates that the higher the value of the metric, the better the tracking performance of the algorithm.

State Fusion	State Prediction	Loss	MOTA↑	IDF1↑	HOTA↑
TSA-FF (40)	LSTM-MP #2	Smooth L1	76.049	78.395	66.591
	LSTM-MP #2	MSE	76.113	78.711	66.468

**Table 7:** Performance of tracking effects for different state prediction models. The symbol “↑” indicates that the higher the value of the metric, the better the tracking performance of the algorithm.

State Fusion	State Prediction	MOTA↑	IDF1↑	HOTA↑
TSA-FF (40)	GRU #2	75.958	78.539	66.206
	LSTM-MP #2	76.113	78.711	66.468
	Kalman Filter (KF)	76.872	79.097	67.528
	LSTM-MP&KF	76.981	79.526	67.705

## Data Availability

The datasets in the experiments are available at https://motchallenge.net (accessed on 8 September 2023).

## References

[1] Li J., Ding Y., Wei H.L., Zhang Y., Lin W. (2022). SimpleTrack: Rethinking and Improving the JDE Approach for Multi-Object Tracking. Sensors.

[2] Vandenhende S., Georgoulis S., Van Gansbeke W., Proesmans M., Dai D., Van Gool L. (2021). Multi-Task Learning for Dense Prediction Tasks: A Survey. IEEE Trans. Pattern Anal. Mach. Intell..

[3] Yang B., Nevatia R. (2012). Multi-target tracking by online learning of non-linear motion patterns and robust appearance models. Proceedings of the 2012 IEEE Conference on Computer Vision and Pattern Recognition.

[4] Huang C., Li Y., Nevatia R. (2013). Multiple Target Tracking by Learning-Based Hierarchical Association of Detection Responses. IEEE Trans. Pattern Anal. Mach. Intell..

[5] Riahi D., Bilodeau G.A. (2016). Online multi-object tracking by detection based on generative appearance models. Comput. Vis. Image Underst..

[6] Mhalla A., Chateau T., Essoukri Ben Amara N. (2019). Spatio-temporal object detection by deep learning: Video-interlacing to improve multi-object tracking. Image Vis. Comput..

[7] Yang B., Nevatia R. (2014). Multi-Target Tracking by Online Learning a CRF Model of Appearance and Motion Patterns. Int. J. Comput. Vis..

[8] Wang Z., Zheng L., Liu Y., Wang S. (2020). Towards real-time multi-object tracking. Proceedings of the European Conference on Computer Vision.

[9] Aharon N., Orfaig R., Bobrovsky B.Z. (2022). BoT-SORT: Robust associations multi-pedestrian tracking. arXiv.

[10] Zhang Y., Wang C., Wang X., Zeng W., Liu W. (2021). FairMOT: On the Fairness of Detection and Re-Identification in Multiple Object Tracking. Int. J. Comput. Vis..

[11] Bewley A., Ge Z., Ott L., Ramos F., Upcroft B. Simple Online and Realtime Tracking. Proceedings of the IEEE International Conference on Image Processing (ICIP).

[12] Wojke N., Bewley A., Paulus D. (2017). Simple online and realtime tracking with a deep association metric. Proceedings of the 2017 IEEE International Conference on Image Processing (ICIP).

[13] Zhang Y., Sun P., Jiang Y., Yu D., Weng F., Yuan Z., Luo P., Liu W., Wang X. (2022). Bytetrack: Multi-object tracking by associating every detection box. Proceedings of the European Conference on Computer Vision.

[14] Ge Z., Liu S., Wang F., Li Z., Sun J. (2021). Yolox: Exceeding yolo series in 2021. arXiv.

[15] He L., Liao X., Liu W., Liu X., Cheng P., Mei T. Fastreid: A pytorch toolbox for general instance re-identification. Proceedings of the 31st ACM International Conference on Multimedia.

[16] Li S., Xiao T., Li H., Yang W., Wang X. (2017). Identity-Aware Textual-Visual Matching with Latent Co-attention. Proceedings of the IEEE International Conference on Computer Vision (ICCV).

[17] Ye M., Shen J., Lin G., Xiang T., Shao L., Hoi S.C.H. (2021). Deep learning for person re-identification: A survey and outlook. IEEE Trans. Pattern Anal. Mach. Intell..

[18] Karanam S., Li Y., Radke R.J. (2015). Person Re-Identification with Discriminatively Trained Viewpoint Invariant Dictionaries. Proceedings of the IEEE International Conference on Computer Vision (ICCV).

[19] Li X., Zheng W.S., Wang X., Xiang T., Gong S. (2015). Multi-Scale Learning for Low-Resolution Person Re-Identification. Proceedings of the IEEE International Conference on Computer Vision (ICCV).

[20] Wang Y., Wang L., You Y., Zou X., Chen V., Li S., Huang G., Hariharan B., Weinberger K.Q. (2018). Resource Aware Person Re-identification Across Multiple Resolutions. Proceedings of the IEEE/CVF Conference on Computer Vision and Pattern Recognition.

[21] Sarfraz M.S., Schumann A., Eberle A., Stiefelhagen R. (2018). A Pose-Sensitive Embedding for Person Re-identification with Expanded Cross Neighborhood Re-ranking. Proceedings of the IEEE/CVF Conference on Computer Vision and Pattern Recognition.

[22] Hou R., Ma B., Chang H., Gu X., Shan S., Chen X. VRSTC: Occlusion-free video person re-identification. Proceedings of the IEEE/CVF Conference on Computer Vision and Pattern Recognition.

[23] Baisa N.L. (2021). Occlusion-robust online multi-object visual tracking using a GM-PHD filter with CNN-based re-identification. J. Vis. Commun. Image Represent..

[24] Wang G., Song M., Hwang J.N. (2022). Recent advances in embedding methods for multi-object tracking: A survey. arXiv.

[25] Chen L., Ai H., Zhuang Z., Shang C. Real-time Multiple People Tracking with Deeply Learned Candidate Selection and Person Re-Identification. Proceedings of the IEEE International Conference on Multimedia and Expo (ICME).

[26] Yang F., Chang X., Sakti S., Wu Y., Nakamura S. (2021). ReMOT: A model-agnostic refinement for multiple object tracking. Image Vis. Comput..

[27] Ristani E., Tomasi C. Features for multi-target multi-camera tracking and re-identification. Proceedings of the IEEE Conference on Computer Vision and Pattern Recognition.

[28] Shen H., Huang L., Huang C., Xu W. (2018). Tracklet association tracker: An end-to-end learning-based association approach for multi-object tracking. arXiv.

[29] Li W., Xiong Y., Yang S., Xu M., Wang Y., Xia W. (2021). Semi-tcl: Semi-supervised track contrastive representation learning. arXiv.

[30] Schroff F., Kalenichenko D., Philbin J. FaceNet: A Unified Embedding for Face Recognition and Clustering. Proceedings of the IEEE Conference on Computer Vision and Pattern Recognition (CVPR).

[31] Feng W., Li B., Ouyang W. (2022). Multi-object tracking with multiple cues and switcher-aware classification. Proceedings of the International Conference on Digital Image Computing: Techniques and Applications (DICTA).

[32] Zhang Y., Sheng H., Wu Y., Wang S., Ke W., Xiong Z. (2020). Multiplex Labeling Graph for Near-Online Tracking in Crowded Scenes. IEEE Internet Things J..

[33] Chu Q., Ouyang W., Li H., Wang X., Liu B., Yu N. Online multi-object tracking using CNN-based single object tracker with spatial-temporal attention mechanism. Proceedings of the IEEE International Conference on Computer Vision.

[34] Zhu J., Yang H., Liu N., Kim M., Zhang W., Yang M.-H. Online multi-object tracking with dual matching attention networks. Proceedings of the European Conference on Computer Vision (ECCV).

[35] Breitenstein M.D., Reichlin F., Leibe B., Koller-Meier E., Van Gool L. (2009). Robust tracking-by-detection using a detector confidence particle filter. Proceedings of the IEEE 12th International Conference on Computer Vision.

[36] Xing J., Ai H., Lao S. (2009). Multi-object tracking through occlusions by local tracklets filtering and global tracklets association with detection responses. Proceedings of the IEEE Conference on Computer Vision and Pattern Recognition.

[37] Chen J., Sheng H., Zhang Y., Xiong Z. (2017). Enhancing Detection Model for Multiple Hypothesis Tracking. Proceedings of the IEEE Conference on Computer Vision and Pattern Recognition Workshops (CVPRW).

[38] Rosello P., Kochenderfer M.J. Multi-agent reinforcement learning for multi-object tracking. Proceedings of the 17th International Conference on Autonomous Agents and MultiAgent Systems.

[39] Jiang X., Li P., Li Y., Zhen X. (2019). Graph neural based end-to-end data association framework for online multiple-object tracking. arXiv.

[40] Weng X., Wang Y., Man Y., Kitani K. (2020). GNN3DMOT: Graph Neural Network for 3D Multi-Object Tracking with Multi-Feature Learning. arXiv.

[41] Wang Y., Kitani K., Weng X. (2021). Joint object detection and multi-object tracking with graph neural networks. Proceedings of the IEEE International Conference on Robotics and Automation (ICRA).

[42] Alahi A., Goel K., Ramanathan V., Robicquet A., Li F.-F., Savarese S. (2016). Social LSTM: Human Trajectory Prediction in Crowded Spaces. Proceedings of the IEEE Conference on Computer Vision and Pattern Recognition (CVPR).

[43] Gupta A., Johnson J., Li F.-F., Savarese S., Alahi A. Social gan: Socially acceptable trajectories with generative adversarial networks. Proceedings of the IEEE Conference on Computer Vision and Pattern Recognition.

[44] Kim C., Li F., Rehg J.M., Ferrari V., Hebert M., Sminchisescu C., Weiss Y. (2018). Multi-object Tracking with Neural Gating Using Bilinear LSTM. Computer Vision–ECCV 2018: Volume 11212.

[45] Sadeghian A., Alahi A., Savarese S. Tracking the untrackable: Learning to track multiple cues with long-term dependencies. Proceedings of the IEEE International Conference on Computer Vision.

[46] Bochinski E., Eiselein V., Sikora T. (2017). High-Speed tracking-by-detection without using image information. Proceedings of the 14th IEEE International Conference on Advanced Video and Signal Based Surveillance (AVSS).

[47] Bochinski E., Senst T., Sikora T. (2018). Extending IOU based multi-object tracking by visual information. Proceedings of the 15th IEEE International Conference on Advanced Video and Signal Based Surveillance (AVSS).

[48] Bergmann P., Meinhardt T., Leal-Taixe L. Tracking without bells and whistles. Proceedings of the IEEE/CVF International Conference on Computer Vision (ICCV).

[49] Bae S.H., Yoon K.J. (2014). Robust Online Multi-object Tracking Based on Tracklet Confidence and Online Discriminative Appearance Learning. Proceedings of the IEEE Conference on Computer Vision and Pattern Recognition.

[50] Brasó G., Leal-Taixé L. Learning a neural solver for multiple object tracking. Proceedings of the IEEE/CVF Conference on Computer Vision and Pattern Recognition.

[51] Bernardin K., Elbs A., Stiefelhagen R. (2006). Multiple object tracking performance metrics and evaluation in a smart room envi-ronment. Proceedings of the Sixth IEEE International Workshop on Visual Surveillance.

[52] Ristani E., Solera F., Zou R., Rita C., Carlo T. (2016). Performance measures and a data set for multi-target, multi-camera tracking. Proceedings of the European Conference on Computer Vision.

[53] Luiten J., Osep A., Dendorfer P., Torr P., Geiger A., Leal-Taixé L., Leibe B. (2021). HOTA: A Higher Order Metric for Evaluating Multi-Object Tracking. Int. J. Comput. Vis..

[54] Yu E., Li Z., Han S., Wang H. (2022). Relationtrack: Relation-aware multiple object tracking with decoupled representation. IEEE Trans. Multimed..

[55] Liang C., Zhang Z., Zhou X., Li B., Zhu S., Hu W. (2022). Rethinking the competition between detection and reid in multiobject tracking. IEEE Trans. Image Process..

[56] Sun P., Cao J., Jiang Y., Zhang R., Xie E., Yuan Z., Wang C., Luo P. (2020). Transtrack: Multiple object tracking with transformer. arXiv.

[57] Gao R., Wang L. MeMOTR: Long-term memory-augmented transformer for multi-object tracking. Proceedings of the IEEE/CVF International Conference on Computer Vision.

[58] Zhou X., Yin T., Koltun V., Krähenbühl P. Global tracking transformers. Proceedings of the IEEE/CVF Conference on Computer Vision and Pattern Recognition.

